# Design and Implementation of a Narrow-Band Intersatellite Network with Limited Onboard Resources for IoT

**DOI:** 10.3390/s19194212

**Published:** 2019-09-27

**Authors:** Zizung Yoon, Walter Frese, Klaus Briess

**Affiliations:** Technische Universität Berlin, Institute of Aeronautics and Astronautics, Marchstr. 12, 10587 Berlin, Germany; walter.frese@tu-berlin.de (W.F.); klaus.briess@tu-berlin.de (K.B.)

**Keywords:** intersatellite communication, nanosatellite network, distributed space system, IoT, M2M

## Abstract

Satellite networks are inevitable for the ubiquitous connectivity of M2M (machine to machine) and IoT (internet of things) devices. Advances in the miniaturization of satellite technology make networks in LEO (Low Earth Orbit) predestined to serve as a backhaul for narrow-band M2M communication. To reduce latency and increase network responsivity, intersatellite link capability among nodes is a key component in satellite design. The miniaturization of nodes to enable the economical deployment of large networks is also crucial. Thus, this article addresses these key issues and presents a design methodology and implementation of an adaptive network architecture considering highly limited resources, as is the case in a nanosatellite (≈10 kg) network. Potentially applicable multiple access techniques are evaluated. The results show that a time division duplex scheme with session-oriented P2P (point to point) protocols in the data link layer is more suitable for limited resources. Furthermore, an applicable layer model is defined and a protocol implementation is outlined. To demonstrate the technical feasibility of a nanosatellite-based communication network, the S-NET (S band network with nanosatellites) mission has been developed, which consists of four nanosatellites, to demonstrate multi-point crosslink with 100 kbps data rates over distances up to 400 km and optimized communication protocols, pushing the technological boundaries of nanosatellites. The flight results of S-NET prove the feasibility of these nanosatellites as a space-based M2M backhaul.

## 1. Introduction

The deployment of IoT technologies and networks, such as NB-IoT (Narrowband IoT) or Low Range Wide Area Network (LoRaWAN), can fully realize their potential when supported by ubiquitous connectivity. While satellite systems alone can provide true global coverage, geostationary communication satellites have long technological cycles (typically 10 years) and are expensive in both development and operation. Thus, small satellite systems are becoming more relevant as a backbone for Internet of Things (IoT) applications and Machine-to-Machine (M2M) communication, in order to cover polar regions, oceans, and rural areas. The integration of a small satellite network into an IoT network will open new possibilities in coping with global challenges such as climate change, pollution, and disaster monitoring.

Established satellite service providers, such as Argos, Iridium, and Orbcomm, have started to provide IoT services to relay ground sensor data by satellite to users. Just recently (2018), several commercial satellite IoT initiatives such as HIBER, DIAMOND, and KEPLER have launched their first nanosatellites, demonstrating the technology and feasibility for satellite M2M communication (see Table 1). These are mostly based on custom Software Defined Radio (SDR) and proprietary protocols.

**Figure 1:**
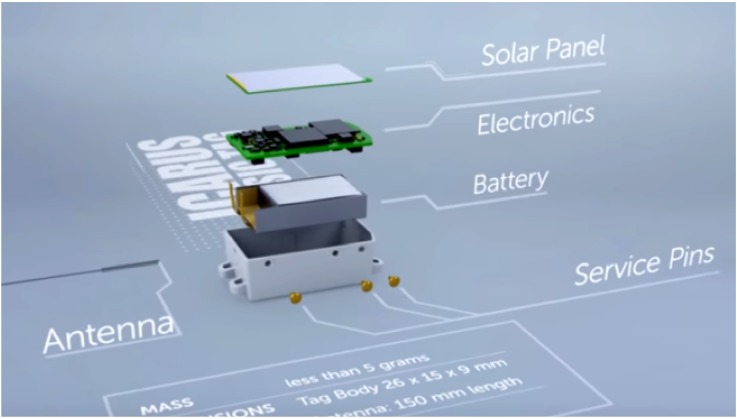
A 5 gr ICARUS animal tag with GNSS, accelerometer, magnetometer, temperature sensor, and 500 MB memory. Credit: Max Planck Society.

**Figure 2:**
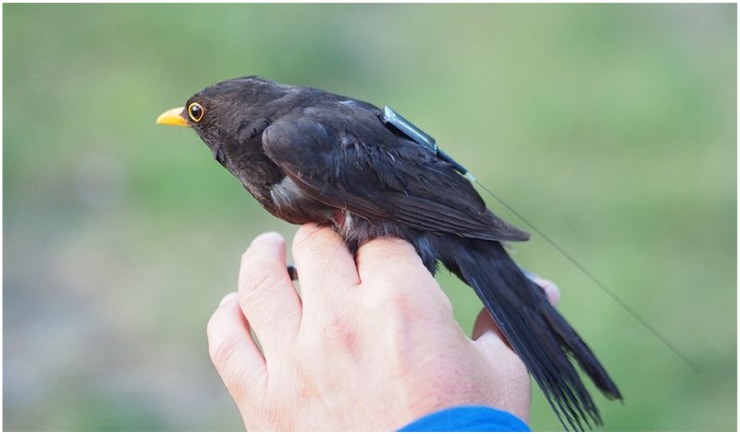
ICARUS tag on a blackbird. Credit: Max Planck Society.

In the scientific domain, the ICARUS system has been installed (in 2018) on the International Space Station (ISS) to establish an internet of animals by enabling animal tracking from space, based on very tiny sensors (Figure 1 and Figure 2). The ISS inclination of 52.6 deg limits the coverage of higher latitudes, however. The system has moderate revisit times for low latitudes (0 to 2 contacts in 48 h at the equator) [1].

The integration of a satellite network into a terrestrial IoT/M2M network is a multi-parameter optimization problem, which includes:

- Type of service: Data rate, bandwidth, latency, QoS, and IoT protocol.
- Regulations: Frequency allocation and space debris removal.
- Space segment: Network topology, coverage (number of satellites, orbit type, and altitude), Inter Satellite Link (ISL) yes/no, antenna type, and beam print.
- Ground segment: Number of ground stations, number of gateways, user terminal Radio Frequency (RF) power and duty cycle.
- Logistics: Launch, operation, and fleet management

This article focuses on the space segment and addresses the design methodology and implementation of an adaptive network architecture with highly limited resources, as is the case in a nanosatellite (≈10 kg) network. A key task for increasing the operational efficiency and shortening the latency in distributed satellite systems is the miniaturization of ISL technology. To push the technological boundaries of ISL miniaturization, a novel and adaptive communication network concept, suitable for low-onboard resource missions, is proposed in this article, which includes possible network topologies for short and medium distances, multiple medium access techniques and channel coding, and an organization of the functional layers.

To demonstrate and verify the ISL feasibility, Technische Universität Berlin (TUB) has developed the innovative mission (S-NET) of four nanosatellites with the S Band (2.0–2.3 GHz) transceiver (SLINK) radio as the main payload [2]. The SLINK, developed at TUB, is the core element of this mission, with 100 kbps crosslink and 1 Mbps downlink rates which make it suitable for accommodation in nanosatellites. On the system level, the ISL data rate mainly depends on the distance, available transmission power, antenna gain, and pointing and is, thus, adaptable. So, higher order of magnitudes can be achieved with more performant platforms. The description of the implemented network architecture and system design of this mission is the main intention of this article. The novelty of this work is the optimization and organization of the functional communication techniques and protocol layers suitable for resource-limited nanosatellites.

## 2. Inter-Satellite Communication

ISL, or crosslink, refers to the capability to transfer data between satellites or a multi-entity network with minimal human interaction. According to the mission purpose, ISL data could be user IoT data from a ground terminal, operational data (e.g., Spacecraft (S/C) relative position data, operation schedule, or housekeeping data), or payload data.

When it comes to the network topology of a multi-satellite constellation, we begin with the optimization problem of coverage, where the criteria are the minimum number of satellites, required revisit time, swath width, and available ISL range. The synthesis of this kind in multi-satellite constellation optimization for Earth discontinuous (periodic) coverage has been thoroughly described in [3,4,5], where Razoumny presented a route theory as a mathematical abstraction for the approximation of an arbitrary satellite constellation.

For global coverage constellations, ISL allows for reduction of the coverage rate of the demand region, as ISL links can be used to route the data from satellites out of the coverage region to satellites that are within the coverage region. Additionally, the number of ground stations can be significantly reduced. In [6], the impact of ISL, with respect to the system throughput of large constellations from Telesat, SpaceX, and OnWeb, has been analyzed. It was shown that, for OneWeb, a hypothetical ISL of 5 Gbps could reduce the number of ground stations from 70 to 27, in order to achieve maximum system throughput; furthermore, the total system performance was 10% higher for the scenario considering 30 ground stations and ISL, as compared with the case of no ISL.

### 2.1. Small Satellite ISL Missions for IoT

The advantages of small satellite constellations with ISL capabilities have been thoroughly discussed and several missions have been proposed; for instance, in [7]. Only a handful of missions, however, have been successfully realized in the past, and were mostly for technological demonstration. Table 2 provides an overview of some notable small satellite ISL missions which have contributed to technological advancement in this field. The majority of past missions used narrow-band UHF links with P2P communication between only two nodes. The robustness and simplicity of UHF technology made it the primary choice for TM/TC in small satellites, from their early development up to present. Despite their limited transmission rate of some tens of kbps, the advantages of low power consumption, very simple antenna design, and cost-effective ground station set-up has made it preferable for universities and research institutions.

The very first mission to demonstrate ISL performance was the SNAP-1 mission from the UK/China in 2002, even though it only demonstrated one-way UHF communication between a nanosatellite and a larger mother-satellite [8]. In CanX-4&5, two satellites were designed to perform formation flight with sub-meter tracking error accuracy. The ISL in this case was, again, a two-way crosslink using S band with an effective data rate of about 10 kbps at distances of up to 5 km [9], in order to exchange position, velocity, and attitude information between the satellites. Admittedly, past missions, such as SNAP-1 or CanX-4&5, did feature an crosslink; however, it was only on a very basic level. These appear insufficient for the envisioned autonomous nanosatellite swarms, with communication distances of at least several tens of km, data rates in the vicinity of 100 kbps, and the capability of multi-hop routing for reasonably effective data exchange.

Recent concepts and missions have implemented more sophisticated ISL techniquesm such as the increase of bandwidth in the S band, extension of communication range, and multi-hop routing protocols. EDSN [10], by NASA, although suffering failure during launch, implemented a multi-hop routing protocol with eight satellites and two Cubesats. The DIAMOND and S-NET missions were among the first nanosatellite missions ever to successfully demonstrate ISL using SDR-based configurable S band radio. Albeit Iridium Next spacecrafts do not fall into the small satellite category; however, they are listed as the Iridium constellation was the first LEO mission to use ISL commercially.

To enable large coverage and short response time for sustainable IoT services, key parameters such as data rate and maximum path length are still constraints. As small satellites are limited in size and power, and as their antenna dimensions are constrained by physics, innovative radio spectrum usage and antenna design must be realized for future operational missions. In particular, in order to enable seamless M2M with terrestrial nodes, a proper IoT protocol which enables hand-off, mobility management, and low power low rate transmission must be implemented. A parameter overview of terrestrial Low Power Wide Area Network (LPWAN) protocols for IoT is given in Table 3. As their transmission power is limited by the regulatory Power Spectral Density (PSD) mask, high RX sensitivity (e.g., −140 dBm for MIOTY^TM^ and antenna gain are required. Alternatively, a gateway between IoT sensors and the satellites could use higher frequencies (e.g., S band) and TX power to mitigate the tight link budget.

## 3. Design of ISL Network for IoT

This section describes the design process of an ISL network for small satellites by identifying and analyzing the communication layer, functionalities, and potential solutions, based on the OSI framework. This includes the physical layer (frequency, bandwidth, channel coding, and modulation), data link layer (medium access control), and network layer (topology and routing).

### 3.1. Design Process

The network design process is driven by a set of design parameters derived from various M2M application scenarios. Some selected high-level design parameters which affect the OSI layer specification are:

- Communication latency: Extending the network to space increases latency, not only due to increased distance but mainly due to link availability. Thus, latency affects orbit design, revisit time, and constellation coverage.
- Power of ground terminal: The M2M ground terminal is constrainted in size, volume, and power (i.e., battery life and solar cell availability). This affects the transmission power, duty cycle, frequency, protocol, and link budget.
- Required bandwidth for Earth-to-Space: The data types that need to be exchanged define the access rates and transmission speed. This affects the selection of frequency, protocol, coding, and modulation.
- Onboard processing power: More processing power is required for centralized topologies and to process a high number of simultaneous IoT links (e.g., CDMA)
- Network topology: Arrangement of satellite nodes. Variable topology allows for flexible orbits and distances among satellites.
- Link connectivity: A continuous link is required for voice communication or safety-related (and, thus, mission-critical) applications.
- Inter-satellite link: ISL can drastically reduce the data forwarding latency, especially for small constellations.
- Reconfigurability/Scalability: The layers should be designed to handle node failures or newly deployed nodes.

A systems design methodology based on the on the Responsive and Formal Design (RFD) approach was applied to the OSI framework in [15]. This methodology has been adapted to identify the possible impact on the OSI layer, depending on various M2M scenarios and system design parameters. The results are given in Table 4, where relevant design parameters (column 1) and possible impacts on the OSI layers (column 2) are identified for different M2M scenarios (columns 3–7). A duplex link allows efficient broadcasting of firmware updates, sensor reconfigurations, or QoS information from space. As an example of reading Table 4, a simplex narrow-band IoT application (e.g., container tracking by a direct ground sensor to the satellite link) is described in column 3. The required communication latency is in the range of minutes to hours, and affects directly the network, data link, and physical layer design for the satellite network.

### 3.2. Frequency and Bandwidth

For ground-to-satellite IoT/M2M communication, the ground devices normally require small data rates (∼kbps), low power consumption for battery life up to several years, compact antenna design, and coarse to even omni-directional antenna pointing. These requirements narrow down the considerable frequency bands for Earth-to-satellite to VHF (0.03–0.3 GHz), UHF (0.3–1 GHz), L band (1–2 GHz), and S band (2–4 GHz).

For the ISL to function as an IoT backbone, the bandwidth depends, besides the required data throughput, also on TX frequency, signal propagation, network topology, onboard resources (e.g., power, size, and pointing), number of simultaneous links, and suitable antennas. Table 5 lists the frequencies currently allocated for ISL and space-to-space communication [16]. While a wide range of frequencies, from VHF/UHF to Ka bands, are usable, most pico- and nano-satellite missions in the past have used the UHF band, due to its omni-directional pattern, robustness, and low power consumption. However, to meet the requirements for an IoT backbone (∼Mbps rate and ∼1000 km range), the frequency selection will go towards the S band, or even the X band. The S band offers a good trade-off between bandwidth, directionality (≈10°), antenna (e.g., patch antenna on S/C), and transceiver size and power consumption.

### 3.3. Channel Coding and Modulation

Basically, two principles of channel coding are used. If the transmission errors are corrected in the decoder, it is referred to as Forward Error Correction (FEC). This approach is suitable for non-optimal channels; the error rate depends on the channel quality, while the data throughput remains constant. Thus, FEC is a better choice for simplex channels (e.g., for payload data downlinks). Error correction is especially recommended for tight link budgets [17]. The second principle is based on Automatic Repeat Request (ARQ), where acknowledgments and timeouts are used to achieve reliable communication. In contrast to FEC, the reliability is constant and the data throughput can vary, depending on the channel quality. For bi-directional data transfer, ARQ can increase the reliability for very low Signal-to-Noise Ratio (SNR)s.

A hybrid scheme of FEC and ARQ seems to be the best channel coding method for nanosatellite networks, for the following reasons: The communication range can vary from some hundred meters up to several hundred kilometers; a truly isotropic antenna pattern is not possible; and RF power is limited and directional antennas rely on the position information of nodes and attitude control to point towards the target. Thus, even though the channel itself has a low noise level, loss-free data transmission is difficult to realize.

**Figure 3:**
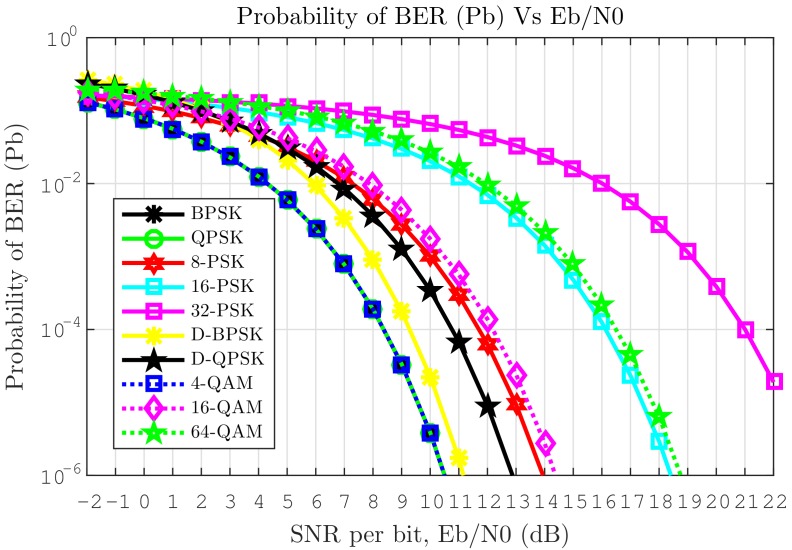
BER versus SNR per bit ($E_{b}$/$N_{0}$) for digital modulation schemes.

Modulation is a trade off between spectral bandwidth efficiency and power efficiency. BPSK requires the least power for a given transmission rate and Bit Error Rate (BER) and is, thus, often the baseline for small satellite TM/TC. QPSK is more bandwidth-efficient; however, it is sensitive to phase distortion on the receiver side. This can be compensated for by DQPSK. Higher order PSK techniques allow for faster data rates at the cost of higher sensitivity, as shown in Figure 3, where $E_{b}$ is the amount of energy received per information bit while $N_{0}$ is the power spectral density of the noise and the BER probability $P_{b}$.

**Figure 4:**
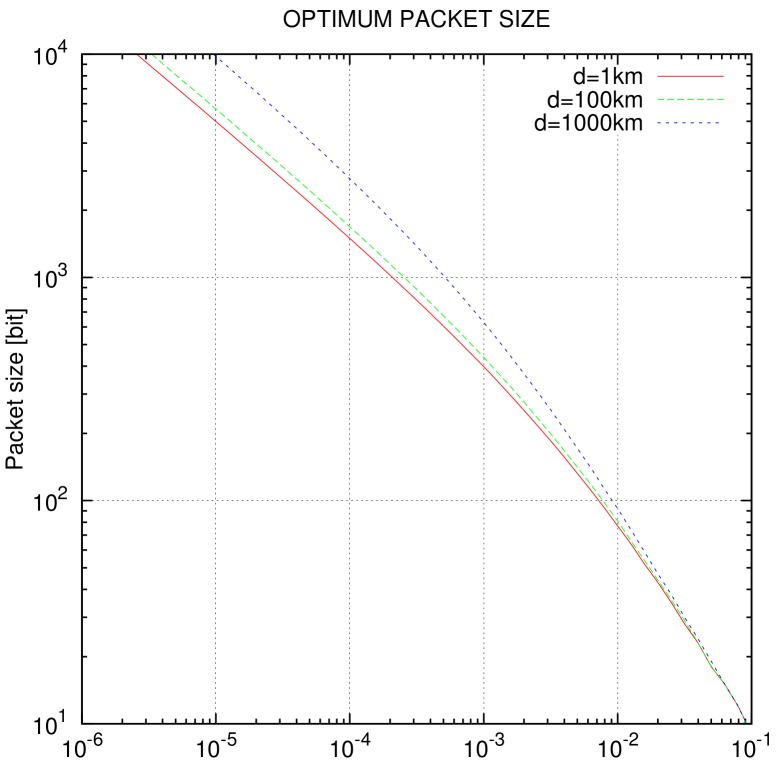
Optimum packet size for QPSK with symbol rate $f_{s}=80\;k$, convolutional coding with coding rate r=3/4, k=9, and BER $P_{b}=10^{-4}$.

The maximum data throughput of an ARQ protocol with respect to the channel robustness (BER) can be simplified when assuming constant signal runtime and protocol overhead, as represented by

(1) $$r_{max}=\frac{n\;\cdot \;{\left(1-P_{b}\right)}^{n}}{n+\alpha },$$

where *n* is the packet size in bits and α is the symbol overhead, determined by the signal runtime and ACK time. The following Equation (2) provides the optimal packet size for given $P_{b}$, in order to maximize the data rate *r*. A respective simulation of *n* for QPSK and different distances is given in Figure 4. The theoretical data rates at BER = 0 for selected modulation TDD distances are shown in Table 6. The data transfer defined by the caller is asymmetrical, as described in Section 3.5.

(2) $$\frac{ln(1-P_{b})}{{\left(1-P_{b}\right)}^{n}}=\frac{\alpha }{n^{2}}.$$

For effective FEC, a terminated convolutional code (Viterbi) with the polynomials (561,753) is used for practical bit-error rates of $10^{-4}$ and lower, 1 dB better than the well-known (171,133) code by NASA. The code rate *r* of 3/4 is constructed through puncturing from the “basic” code with r=1/2. A soft-decision Viterbi decoder is used for error correction. The optimal packet size depends on BER $P_{b}$, and is simulated for QPSK in Figure 4.

### 3.4. Medium Access Control

The Media Access Control (MAC) layer is the lower sublayer of the data link layer (layer 2) in the Open Systems Interconnection Model (OSI) model. Technical feasibility of hardware (antenna), application requirements, network topology, available bandwidth, and the number of nodes are the main criteria for selecting a suitable channel access method for a satellite network. This section provides a short analysis of common multiple access protocols, such as TDMA, FDMA, CDMA, and SDMA, with respect to their suitability for nanosatellite networking. Additionally, a short analysis on packet switching methods—namely, random access and token passing procedures—is provided. Space Division Multiple Access (SDMA) is not analyzed, due to large and complex antennas (spot beam and phased array antennas) required for a nano-spacecraft and the strict requirements for attitude control. A good overview of the requirements for antennas and corresponding concepts has been given in [7], which is adapted in Table 7. The current technology allows the use of reconfigurable phased antenna arrays even for Cubesats, as proposed in [18].

FDMA and CDMA have the advantage that multiple crosslinks can be established simultaneously. In the case of FDMA, the most problematic issue is that, with an increasing number of nodes, the required bandwidth increases as well, as one center frequency is required for each cross-link. S Band frequencies are extensively used, at present, for TM/TC services and thus may cause regulatory conflicts. Furthermore, the use of FDMA leads to a significant increase of complexity and cost of the communication system, due to the necessity of extensive band-pass filtering. On the other hand, CDMA requires quick and precise RF power control to limit interference, as well as complex signal processing. Satellites, particularly those within different orbits, can have large relative velocities, which may result in a strong variation of signal strength in the decoder and, thus, make safe signal separation difficult. FDMA and especially CDMA would be good choices for PFF missions with a small number of S/Cs and very strict time latency requirements, as proposed in [19].

For small formations, synchronous TDD can be used (classic TDMA). The TDD method assigns time slots to each node; therefore, a single frequency is sufficient for all cross-links, which allows for the replication of each node, in terms of frequency and bandwidth. The constraints of TDD are that time synchronization among the nodes is needed and, for a large number of S/C, the duty interval for transmission increases, thus resulting in lower data throughput. Furthermore, the time slots limit the allowable propagation delay and, thus, the distance between satellites.

For S-NET missions, synchronization is carried out by ground command. A dedicated master S/C is used for time synchronization and control of the network. This asymmetrical organization of function/responsibility, however, leads to higher resource consumption in the master. Additionally, two channel access methods based on asynchronous TDD rules for packet switching will be demonstrated: Token passing and random access. In both cases, P2P protocols are used while only one P2P connection is allowed at a time. Bi-directional P2P data transfer is time-duplexed and organized in short sessions of variable length. Each session consists of a number of TDD frames. In this context, suitable channel coding is important (see Section 3.3). The token passing scheme is collision-free and simple to implement, and is thus a suitable choice for formations with a small number of nodes. For a line topology, the token is passed back and forth from one end to the other and, hence, a routing algorithm is not required. For a fully connected topology, a token ring organization is suitable.

**Figure 5:**
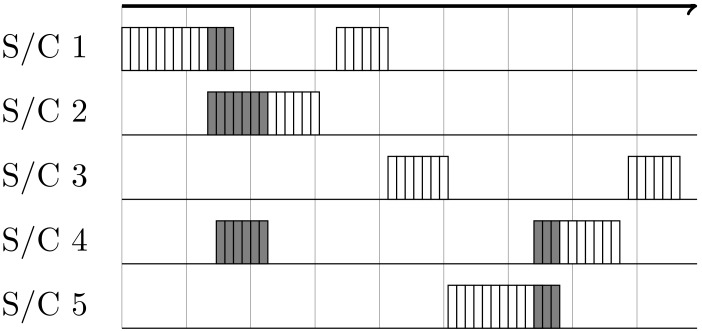
Simplified diagram of random channel access. Boxes indicate short bi-directional sessions with flexible or fixed lengths. Shadowed boxes indicate session frames which have collided.

For a higher number of nodes and multi-hopping, the more advanced random access method is preferable, due to higher data throughput while allowing package collision. Even simultaneous links on the same channel are possible, if proper spacial separation can be achieved. As each session consists of several TDD frames, only collided frames are lost in the case of a collision (see Figure 5). This scheme can be extended by a collision avoidance rule and/or a power control technique.

### 3.5. P2P Duplexing Scheme

A data link layer (OSI layer 2) must incorporate rules or procedures for link establishment, for lossless data transfer, and for terminating communication. A P2P protocol and TDD seem to be a suitable solution for nanosatellite networks and perform well; particularly in a meshed topology where multi-hopping is unavoidable and no central controller exists.

**Figure 6:**
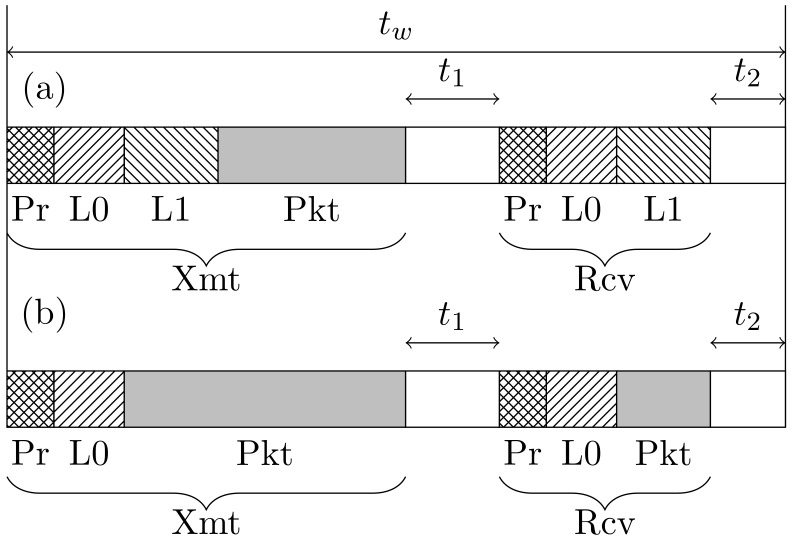
TDD frame structure: (**a**) Link establishment and adjustment phase; and (**b**) data transfer phase (with ARQ possible in one direction only).

The two nodes of a P2P link are referred to as the *caller* and *responder*. A caller satellite is the initiator of the session establishment process. A responder satellite receives session establishment parameters from the caller. A session is a continuous dialog between the caller and responder and consists of three distinct phases: Session establishment, data services, and session termination. Figure 6 illustrates the TDD frame structure for: (a) Link establishment and (b) data transfer. The P2P bi-directional TDD data stream is divided into frames with constant length $t_{W}$. Each frame contains two subframes: One caller frame Caller frame (Xmt) and one responder frame Responder frame (Rcv). The data transfer is asymmetrical: Xmts are longer than Rcvs, which typically consist only of an ARQ or control information. Each Xmt consists of a Preambel (synchronisation field) (Pr) for one-shot packet frame synchronization, one or two headers ($L_{0}$/$L_{1}$), and one data block Data packet (information field from network layer) (Pkt); Rcvs have a similar structure. The time $t_{1}$ between Xmt and Rcv is clearly equal to the two-way propagation time $2\times t_{p}$. To keep the frame length constant, $t_{1}+t_{2}$ is chosen to be slightly longer than the maximum time required to communicate over the greatest distance.

The so-called zero layer header $L_{0}$ contains 12 BPSK modulated and convolutional coded data bits with a code rate of r=1/2. The $L_{0}$ header provides a frame counter for the ARQ procedure and data direction information.

The $L_{1}$ header is used for: (i) Session establishment; and (ii) adaption of the modulation and coding scheme during data transfer. Thus, the modulation and coding scheme can be altered quickly within the session, depending on the channel condition, by $L_{1}$. Parameters such as S/C caller and responder IDs, as well as modulation and coding options are also provided by $L_{1}$. The caller and responder IDs are especially relevant for routing in the network layer. $L_{1}$ includes 31 bits, where the modulation and coding are the same as in the case of $L_{0}$ to guarantee a low bit-error probability in bad channels.

Once a session is established and the channel is stable, no $L_{1}$ header is necessary, and more bandwidth can be provided for data packets by the data transfer frame, as illustrated in Figure 6b. The responder answers during the data transfer phase with $L_{0}$, which is detected by the caller as an Acknowledge (ACK). If no ACK can be correctly decoded by the caller within the time $t_{1}+t_{2}$ (Negative Acknowledge (NAK)), Pkt will be retransmitted in a new Xmt frame. The ARQ protocol follows the classic stop-and-wait procedure. A terminating rule is implemented in the case that only NAKs are detected and the SNR is not good enough to guarantee an orderly data transfer on the lowest modulation/FEC combination.

The modulation and coding scheme can be quickly changed within a few TDD frames, depending on the current SNR. The SNR detected by the responder is transmitted in each Rcv frame. After receiving a few Rcv frames and building an average SNR value, the caller makes a decision, based on responders and his own SNR, about the necessity of changing the modulation and coding scheme. Then, an adoption is performed by the caller, which sends a $L_{1}$ header within the next TDD frame.

### 3.6. Network Model

**Figure 7:**
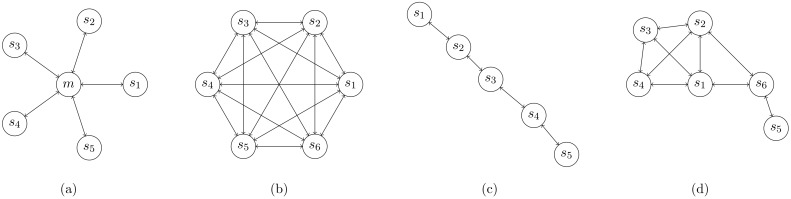
Satellite network topologies: (**a**) Star; (**b**) fully connected; (**c**) line; and (**d**) mesh.

Herein, a satellite network is defined as a set of nodes that are able to communicate over wireless links. The nodes are characterized by their high mobility, as not only fixed satellite constellations but also autonomous orbital maneuvers, reconfiguration, and swarm behavior must be considered. This means that the link availability between a particular set of two nodes is, generally, time-variant. Thus, a satellite network is usually a meshed network (see Figure 7d), which needs to be controlled by a distributed algorithm. An important design baseline is that all nodes (satellites) in a network are technically identical and have the same resources onboard; most notably, they have the same electrical energy. Hence, it is not advantageous to distinguish between routers and user terminals.

Compared to a classical ad-hoc sensor network, a satellite network has some significant differences. First of all, in ad-hoc or wireless sensor networks, two of the most important determinant conditions are inavailability of the position information of the wireless nodes and high and unpredictable mobility of the nodes. This causes a necessity for highly dynamic collection of routing information, inevitably decreasing the data throughput. However, especially for satellite networks, the node positions (and, thus, their availabilities) are highly predictable. Hence, a meshed nanosatellite network has the following characteristics:

- The positions of all nodes at each moment are known; indeed, orbital mechanics allow for good prediction. Orbital maneuvers may lead to changes in the physical network topology. However, they are slow compared to the changes in signal quality and the connection matrix of terrestrial wireless meshed networks, where not only the movement of the nodes, but also wave propagation effects (such as unpredictable shadowing and reflection) play a significant role.
- Significantly great distances, up to 400 km and more; consequently, signal propagation time has a large impact on the timing scheme and MAC.
- Clearly, more resources are available onboard nanosatellites (several Watts, 32 bit μC, several MB of RAM), compared to a node of an ad hoc sensor network (often primary cells, 8 bit μC, few KB RAM).
- Line-of-sight (LOS) wave propagation is characterized by no shadowing and almost no reflection, scattering, or diffraction; so the channel can be well approximated with the AWGN model.
- For multiple orbit constellation, considering that highly inclined SSO are the first choice for small satellites, the formation will be “stretched” over equatorial regions and will be more compact above the poles; thus, regular changes in edge number and weights are expected.

### 3.7. Network Topology

Typically, the network topology is determined not only by the orbital configuration, but also by the network operating mode, desired QoS, and available resource differences between the nodes. The orbital configuration is the most heavily weighted factor affecting the basic network topologies. The simplest configuration consists of only very few S/Cs placed into the same orbital plane with short intersatellite distances, as in the case of Precise Formation Flying (PFF). In this case, the communication graph is fully connected. It can, thus, be approximated with a fully connected mesh (see Figure 7b) or, if one satellite is used as a base station (sometimes called the master), with a centralized star topology (see Figure 7a). As each pair of nodes can be interconnected directly or through a master S/C, no advanced routing techniques are necessary. In the case of the star topology, the channel synchronization can be organized by the master S/C. With a fully connected scheme, either a satellite with a master function is chosen (arbitrarily or by the ground station), or a random access protocol (e.g., pure ALOHA) can be used.

Another common occurence is when the satellites are placed into the same orbital plane with separation distances approximately equal to the maximal possible communication range (i.e., a line topology; see Figure 7c). The most common and difficult case is the meshed topology (see Figure 7d), where the graphical representation of the network can change over time. The control algorithm of this topology is relatively difficult, because no centralized master can control the routing, and multi-hopping is necessary. However, such an algorithm has an important feature: It can be used to control each of the other above mentioned topologies, albeit not always with the optimal performance.

### 3.8. Network Routing

In the cases where multi-hop communication is required to connect particular nodes, flooding techniques provide an option for unicast connections, as well as for broadcasting. However, particularly for large distances, flooding algorithms perform worse, compared to routing ones, due to duplicated packages and higher bandwidth load; hence, they are not considered further in this paper.

A routing protocol is called proactive when the routing information (destination and routes) is stored in the memory and the routing table is updated periodically throughout the network. Constantly updating the routing tables due to topology changes when no traffic is being transmitted may be considered wasteful when there are limited resources. In comparison, in a reactive routing protocol, new routing information must be identified each time in order to transmit a new message, which generates extra overhead. As the topology of a satellite network is predictable, no overhead typical of reactive routing is necessary. However, the routing tables must be dynamically updated as topology changes are identified.

A memoryless distributed algorithm based on unicast geographic routing is, thus, recommended. No control information (e.g., routing tables) needs to be exchanged between nodes, and no central controller is required. Let G=(V,E) be an Euclidean three-dimensional surface consisting of a finite number of vertices *n*. The task of a geographic unicast routing algorithm A is to find an optimal path from any source *s* to any defined destination *t*, while complying with the following conditions:

- All nodes v∈V know their own geographic positions and the geographic position of all other nodes w∈V∖v.
- There is no control information which needs to be stored in the node (i.e., it is a memoryless algorithm).
- Nodes are not allowed to change any information addressed to another node.
- Nodes are not allowed to maintain any information addressed to another node, except for temporary storage before forwarding.

Dijkstra’s algorithm fulfills these requirements [20]. In addition, Dijkstra delivers an optimal solution and has a finite running time, and was thus selected as an optimal algorithm for meshed satellite networks. It performs well in every discussed network topology with connected graph representations (see Figure 7). Each node’s position information can be updated, by ISL or TM/TC, into the onboard orbit prediction algorithm.

Dijkstra’s algorithm scans all vertices with distances smaller than dist(s,t) and is, thus, slower for larger graphs. With a high number of nodes, goal-oriented techniques, such as the A* search or Arcflag, can accelerate the point-to-point computations significantly, as proposed in [21]. If backwards search is required within complex graphs to increase robustness, the SHARC algorithm provides a fast and robust unidirectional routing method [22].

## 4. Mission S-NET

To demonstrate the above proposed network concept, the nanosatellite based mission S-NET was designed and implemented. The following sections describe the mission architecture.

### 4.1. Mission Overview

With increasing numbers of communication entities participating in the network, the network protocol and hardware can be tested in more complex scenarios. For a demonstration mission, four satellites present the best cost–benefit ratio. Four entities enable six independent communication links, while three satellites would only make three links possible. Furthermore, multi-hop communication can only be realized with at least four nodes if they are in a line topology (as in Figure 7c). Additionally, four satellites allow for redundancy on the satellite level, as ISL can also be performed, with some restrictions, with only three satellites.

The S-NET satellites were inserted into a 580 km SSO by a single upper stage and ejected from dispensers with a short time separation. To keep the space segment simple, a propulsion system was intentionally omitted and, therefore, the relative formation is mainly controlled by the separation parameters from the launcher upper stage. The goal is to keep the satellites in a range to enable a stable ISL for at least four months. Therefore, the separation direction, angle, and sequence are the initial values for the formation drift. A summary of the mission parameters is provided in Table 8.

### 4.2. Fault Tolerant Satellite Bus TUBiX10

**Figure 8:**
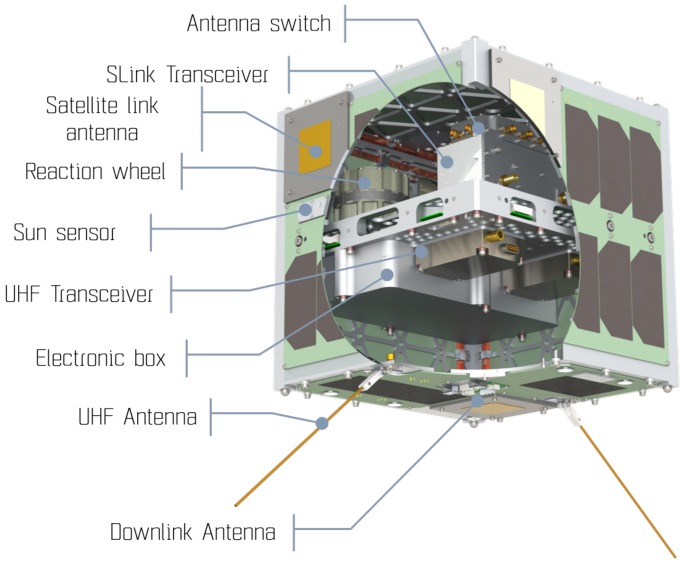
View of the TUBiX10 nano satellite bus.

The nanosatellite platform TU Berlin innovative neXt generation bus (TUBiX) 10 aims for a high integration level to obtain the maximum bus performance within the given form factor. The structure of the TUBiX10 is designed to specifically fit the dispenser SNL. The cube shaped structure, with 25 cm edge length, features four guiding rails parallel to the separation direction to avoid tilting during the ejection process (see Figure 8). A mechanical specification has been developed to adapt future TUBiX10-based satellites [23].

**Figure 9:**
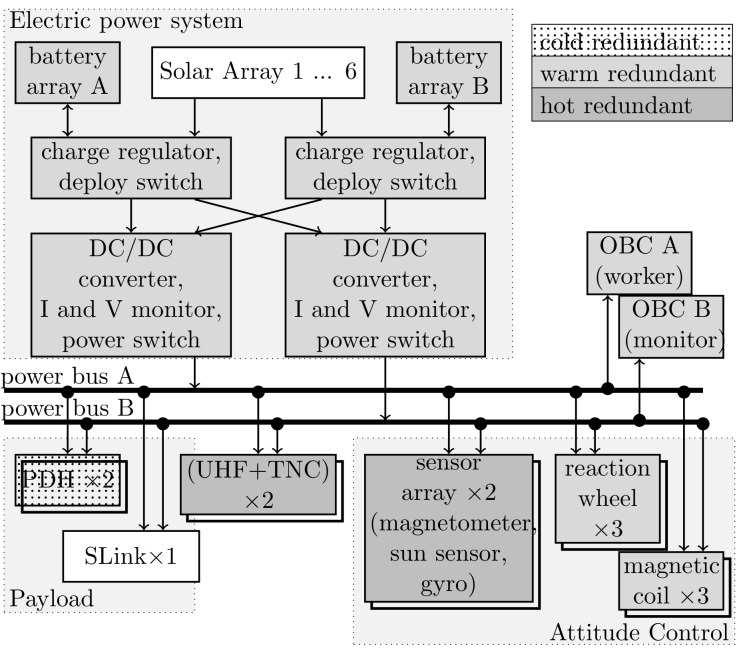
Redundancy and power distribution of space segment.

The middle deck accommodates the battery and backplane, and additionally serves as a mounting interface for the electronics box, payload, reaction wheels, and communication modules. The side panels are also electrically and mechanically connected to the middle deck. Despite the fact that four satellites provide redundancy on a satellite level, the bus is designed to be single-failure tolerant on the subsystem level. Thus, the main subsystems OBC, EPS, PDH, and ADCS are fully redundant. Figure 9 shows an overview of the single-failure tolerant bus architecture.

The power string, including the batteries, battery control, power switches, and DC/DC converters, is completely redundant. The six solar generators are connected in parallel. Each generator consists of eight solar cells and a diode for the prevention of current back-flow connected in series. Triple junction GaAs solar cells, with an efficiency of more than 30%, are used for energy generation. No sun pointing is required for a positive energy budget in nominal operation. The complete system allows for a peak power consumption of approximately 40 W, with 4.5 W on average. The maximum power generation, in the best case, is approximately 7 W.

The Communication System (COM) consists of two UHF transceivers, which are operated in a hot redundant configuration and are permanently in RX mode attempting to decode ground commands using omni-directional antennas. The Terminal Node Controller (TNC) connects to the UHF transceiver and performs modulation/demodulation and coding/decoding. The main payload SLINK is not redundant and can be powered on for the duration of a satellite pass, either to perform ISL experiments or to transmit the experimental data to ground.

### 4.3. S Band ISL System

The core components of the ISL system are the SLINK radio, the Spacecraft Network Controller (SNC), and the antenna system. SLINK accommodates the physical layer and data link layer (OSI layer 1 and 2). The SNC provides the higher communication layers and is responsible for the routing, control, and data logging, in particular. The antenna switch toggles all six ISL antennas to power the correct patch antenna during an ISL session.

#### 4.3.1. Functional Organization

To maintain adaptivity for various missions, it is important to clearly define the functional interface between the ISL terminal and the controller, represented by the SNC. A suitable interface description is recommended in the Proximity-1 protocol, which was proposed by CCSDS for planetary missions where autonomous communication terminals are used between the lander and orbiter [24]. The S-NET mission has adopted the suggested functional distribution. The ISL radio contains a physical layer (OSI layer 1) and a data link layer (OSI layer 2). The mission-specific network and routing algorithm (network layer 3), which is based on Dijskstra’s algorithm, is implemented within the SNC. The responder ID of the $L_{1}$ layer (see, also, Section 3.5) is checked, at this instance, to perform proper routing and transportation of the message. To support the memoryless routing rule, the network layer must also determine the geometric relations within the network, normally done by an orbit propagator (e.g., Simplified General Perturbations (SGP)).

**Figure 10:**
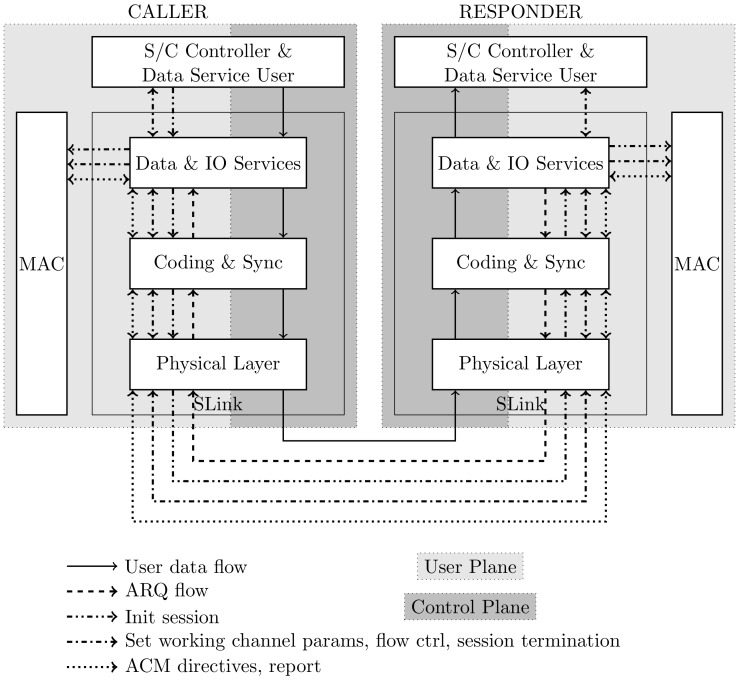
Flow of data and messages between transceiver and controller.

Two kinds of data units are used for data exchange between the SNC and the ISL terminal. One data unit has a variable frame length and is used for the exchange of unspecified service data provided by the data service user. The other unit is reserved for the exchange of user and control information, typically generated by the network layer (see Figure 10).

#### 4.3.2. S Band Radio

**Figure 11:**
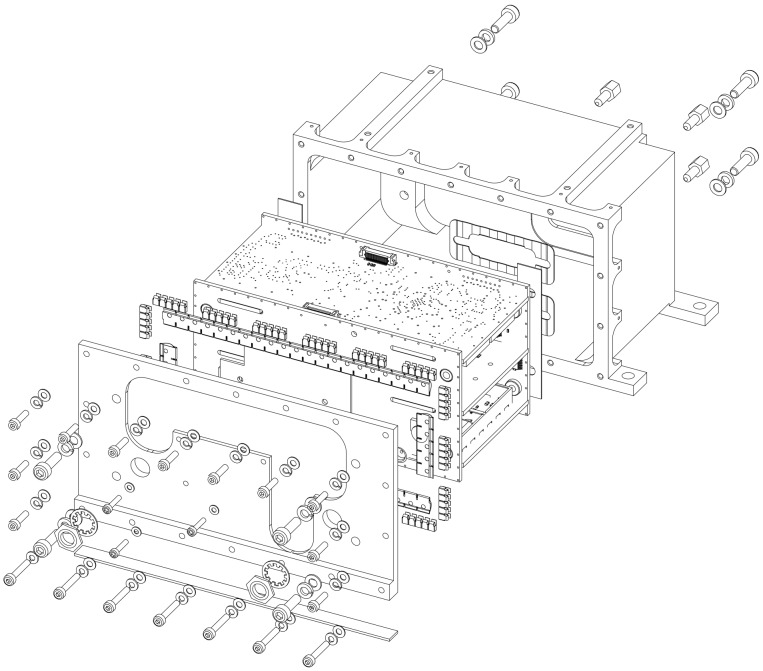
View of S band radio SLINK for ISL, UL, DL, and antenna switch.

TUB, in co-operation with IQ wireless Ltd., has developed a highly integrated S band transceiver (SLINK, see Figure 11 for an overview and Table 9 for the parameters) with a maximum of 169 kbps crosslink capability suitable for nanosatellites. SLINK offers two operating modes, selectable by SNC: An up-/down-link mode, and an ISL mode. The downlink is characterized by 1 Mbps at a symbol rate of 1.4 MHz, the uplink by 32 kbps, and the ISL by 100 kbps nominal. Both ISL and UL have a symbol rate of 80 kHz. The use of phase modulation and effective channel coding (convolutional) lead to a good SNR, with only a small transmission power of 27 dBm possible. The ground link is designed for a 3 m ground station reflector at a minimal elevation angle of 10 deg at *LEO. The nominal downlink data rate of 1 Mbps can be improved by adapting the bandwidth—QPSK and 8PSK are optional—or the channel coding, with variable code rate of 0.25–0.75. In contrast, the uplink bit rate is constant and better than 32 kbps. The up- and down-links are separated in frequency (2.0 and 2.2 GHz, respectively) and in time.

#### 4.3.3. Antenna System

**Figure 12:**
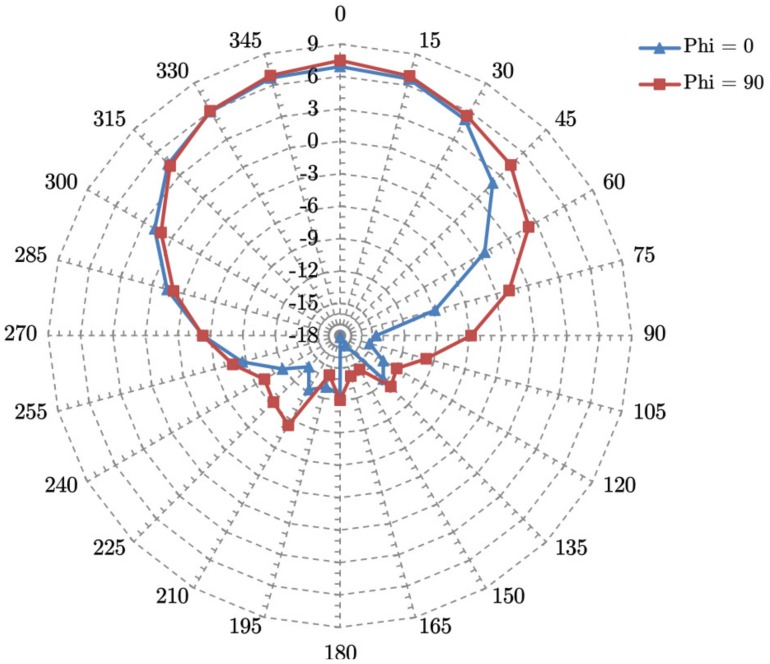
Horizontal (phi = 0) and vertical (phi = 90) antenna pattern of the ISL and downlink antenna.

**Figure 13:**
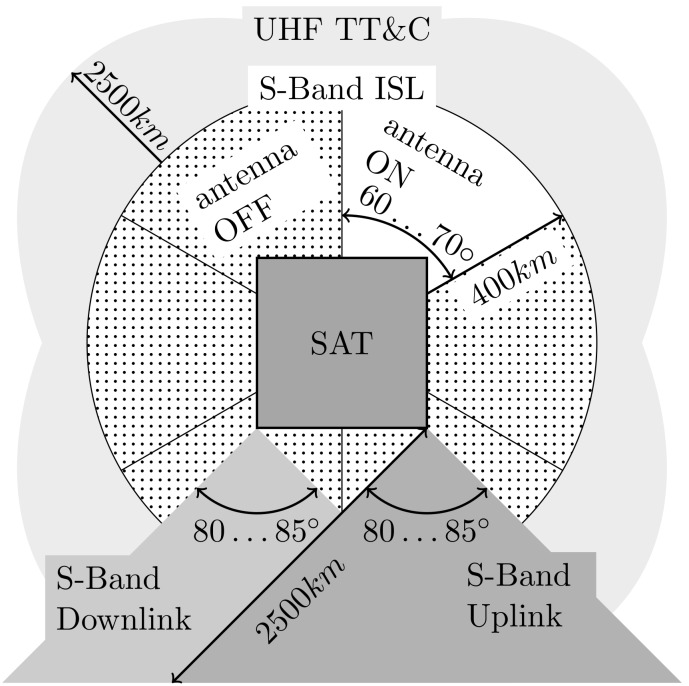
Antenna characteristics for UHF and S band communications.

Two circular polarized directional planar antennas are used for down- and up-link. Five additional planar antennas with a beam pattern of approximately ±30° (pattern shown in Figure 12; nominal gain 6 dbi), one mounted on each non-nadir surface, are responsible for the ISL. Limited onboard resources prohibit activating all ISL antennas simultaneously. Instead, an active antenna switch unit is connected to all ISL antennas. The line-of-sight antenna pair is selected by a scan algorithm, where the caller and responder antennas are switched consecutively until a match is detected. Alternatively, if position and attitude information of both caller and responder are available, the line-of-sight antenna pair can be analytically estimated by orbit propagation, thus shortening the build-up delay. Once a session is established on the physical layer (OSI layer 1), attitude control must be used to keep the line-of-sight stable within the antenna field-of-view. Figure 13 illustrates the antenna concept for the ISL and also the UHF TM/TC channel. The corresponding link budgets for 10, 65, 148, and 400 km ISL distances are summarized in Table 10.

#### 4.3.4. Spacecraft Network Controller

The SNC implements the higher layers, including the network layer (routing algorithm). An orbit propagator (SGP4) is implemented to estimate the position of each node, which are the input parameters for the geometric routing algorithm (see Section 3.8). The communication interface to the main OBC, which generates user data, is realized by SPI. Data communication with SLINK is implemented by SPI for data frames and RS485 for data frames and control information. In order to perform various ISL routing methods, as proposed in Section 3.8, a state machine within the SNC stores control sequences to command the SLINK terminal. Physically, the unit is based on a 32 bit controller, including a latch-up protection circuit, an external watchdog, and low-voltage protection.

## 5. Flight Results

### 5.1. Network Topology

**Figure 14:**
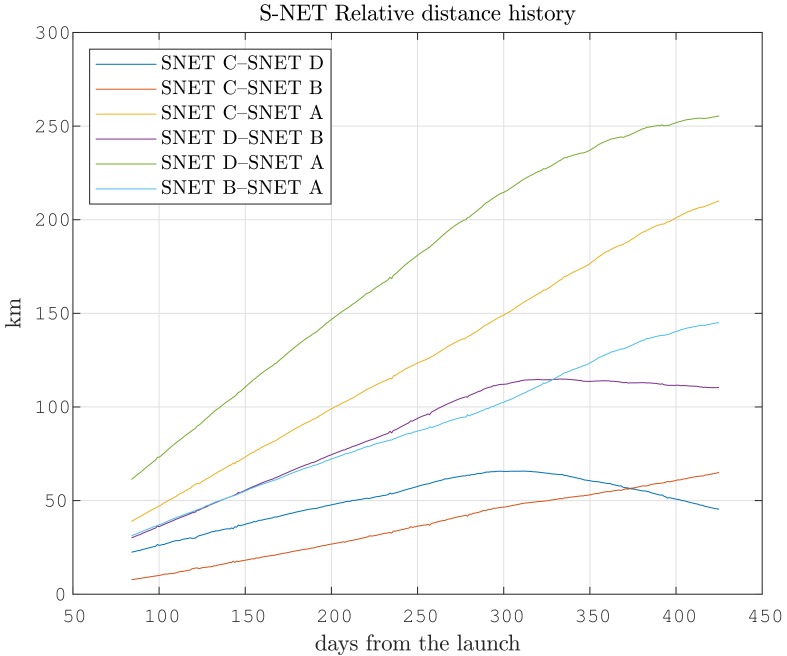
Relative distance development of satellites since launch.

The network topology depends on the physical distances of the satellites. The relative distance of the distributed mission relies on the initial deployment parameters, orbital perturbations, and atmospheric drag control. Figure 14 shows the relatively stable distances among the nodes with a sub-kilometers drift per day, as they were placed into the same orbital plane. The drift reduction, starting at approximately day 300, is the result of a phasing maneuver by active drag control.

**Figure 15:**
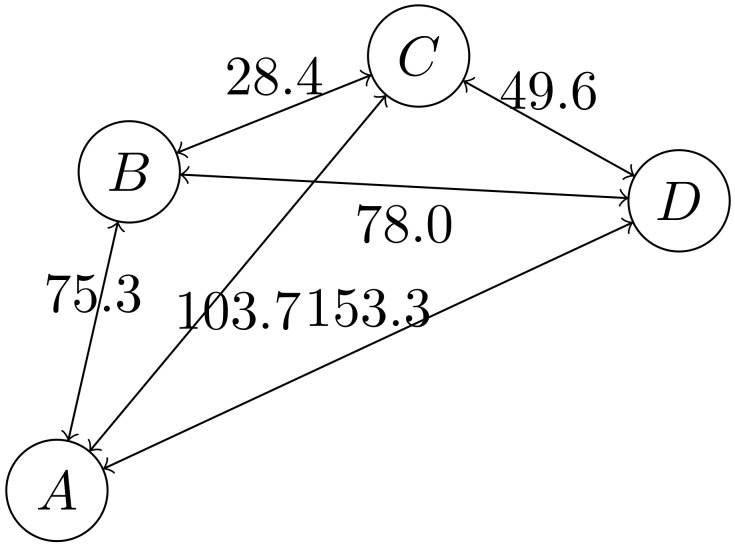
Graph representation of S-NET nodes (August 2018, not to scale) with numbers in km.

The network topology is determined by: (i) The orbital configuration; (ii) the satellite operating mode and desired QoS; and (iii) possible resource differences between the satellites. The orbital configuration is the most heavily weighted factor affecting the basic network topology. In a first-order approximation, the edge weight is linearly down-scaled from the propagation distance. A time snapshot of the communication graph is given in Figure 15. This case refers to a complete graph, since the maximum distance of 400 km was not exceeded. Each pair of nodes could be interconnected, either directly or through a master node, without multi-hop routing.

### 5.2. Link Budget Verification

**Figure 16:**
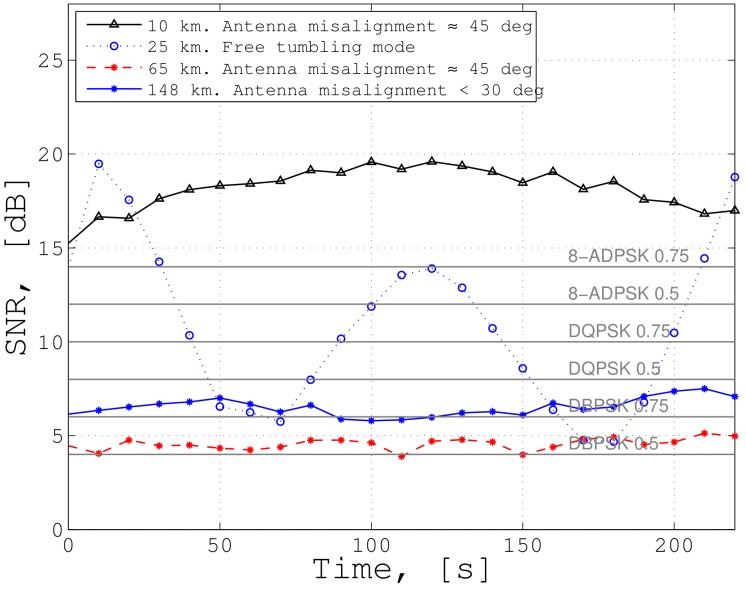
Measured SNR for various distances and pointings within an ISL session [25].

In order to verify the link budget given in Table 10, in-orbit measurements of SNRs for various distance and directional alignment were performed. Due to regulatory constraints, the ISL sessions were only established while in contact with the ground station. The results are depicted in Figure 16. The measurements were done for distances of 10 km (45° pointing offset), 25 km (no attitude control, thus free-tumbling), 65 km (45° pointing offset), and 148 km (30° pointing offset). The x-axis shows the duration for the corresponding ISL session. The antenna pairs remained unchanged during a session. The black horizontal lines indicate the lower limit for each modulation scheme. For BPSK (r=0.5), a SNR of 4 dB is required; for 8-PSK (r=0.75), a SNR of 14 dB is required.

For 65 km distance and a constant pointing offset of 45 deg, the SNR remained stable, at an average of 4.6 dB, which aligns with the theoretical margin of 4.69 dB from Table 10. For 148 km distance, the measured average SNR margin of 7 dB corresponded well to the theoretical value of 7.56 dB. One source of discrepancy between measured and analyzed SNR might the attitude determination of the ADCS, which induces biased values for the RX and TX gains. The strong SNR variation in the 25 km free tumbling case resulted from the pointing loss due to uncontrolled rotational motion. The limit of 400 km will be tested as soon as this distance is reached.

### 5.3. Synchronization and Timing

When establishing an ISL session, both the caller and responder unit must synchronize before exchanging data frames. The sync procedure is the dominant source of latency, and contains the Automatic Gain Control (AGC) (and optionally the Antenna Search Algorithm (ANS)). During AGC, the responder performs a gain sweep (800 ms for one sweep) and tries to detect preambles sent by the caller. Once a preamble is detected, it starts transmitting a reply frame to the caller unit. Simultaneously, the caller performs a RX gain sweep (320 ms for one sweep) to identify the preambles send by the responder. Sync is achieved when both units have detected preambles from the counterpart (see, also, Section 3.5 for the frame structure). Additionally, ANS is performed if the proper antenna pair is unknown prior to the ISL session. In this case, an antenna switch is done after each AGC sweep.

**Figure 17:**
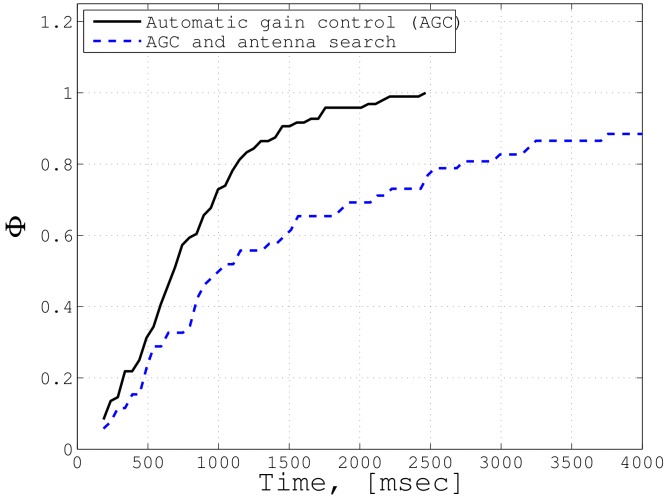
Cumulative distribution function of sync time during ISL sessions [25].

Figure 17 shows the cumulative distribution function for the sync timing of an ISL session in orbit. In 90% of cases, a P2P connection could be established within 1500 ms using AGC only. When using AGC and the optional ANS together, 90% of synchronization was established within 3500 ms. For dedicated IoT/M2M missions with near-real-time latency requirements (∼s), the sync time can be further shortened by reducing the sweep steps of AGC. The ANS can be alleviated by determining the proper antenna pairs prior to each session, which requires knowledge in attitude and orbit position of each pair. By this means, overall P2P latency of less than a second is achievable. For IoT applications with moderate latency requirements (i.e., greater than a few minutes), the sync time carries no weight. Thus, from a timing perspective, all scenarios presented in Table 4, except for the real-time applications (≪1 s), such as voice communications or co-operative driving, can be supported by this technology.

## 6. Conclusions

The integration of a satellite network into a terrestrial IoT/M2M network is a multi-parameter optimization problem. This article identifies the key ISL technologies to enable an effective, fast-responding, and cost-effective narrow-band satellite network which can support M2M applications and services. Some significant advantages are a decrease of communication latency, a reduction in the number of ground stations, and an effective reconfiguration ability of the fleet. In this article, we focused on the space segment and addressed the design methodology and implementation of an adaptive ISL network architecture with highly limited resources. System design parameters and related communication layers for a network with extremely limited on-board resources, suitable for the case of nanosatellites, were analyzed. This included the analysis of appropriate methodologies and techniques for the communication layer, such as routing algorithm, medium access control method, channel coding, and modulation.

These techniques were implemented into a software-defined S Band radio (SLINK), incorporating the physical layer and data link layer. The communication layer between tge SLINK radio and SNC were defined according to the CCSDS recommendation; thus, the network layer was implemented on the spacecraft side. Using TDD, adaptive modulation up to 16-ADPSK, and convolutional code with FEC and ARQ, the equipped version achieved a 100 kpbs data rate over 800 km distance, pushing the state-of-the-art performance for nanosatellite communication. Adaption of the TDD protocol for distances more than 2000 km is easily possible.

To verify the ISL performance and demonstrate system capability in orbit, a fault-tolerant nanosatellite mission S-NET, consisting of four satellites, was developed by TUB. The four S-NET satellites were launched on 1 February 2018 by a Soyuz/Fregat launcher from Vostochny Cosmodrome, Russia, into a 580 km orbit. Since then, the overall performance of the SLINK radio, the link budget and timing of ISL synchronization, data routing, and long-term stability have been successfully verified in the space environment. For this specific mission, the ISL range of 400 km was limited by the link budget, mainly dictated by the TX power and antenna gain. The S-NET mission is, thus, one of the first nanosatellite missions to perform multi-point network communication in orbit.

For the purposes of integration into an IoT/M2M network, the overall ISL performance, such as data rate, distance, and latency, must be adapted to specific scenarios and the resulting system architectures. The number of nodes can be scaled up by adapting the addressing part of the TDD frame header and extending the routing algorithm. Data throughput and ISL range depend on the link budget; hence, efficient spectrum usage and innovative (deployable) antenna concepts are required. The latency performance seems to be compliant with most IoT applications, as the ISL synchronization time for a P2P session can be trimmed to under 1 s. The additional delay for uplink (ground device to sat) and downlink depends on the link design and terrestrial IoT protocol used, but are typically in the range of several seconds. hence, the overall latency—from IoT ground sensor to gateway (optional) to satellite (multi-hop) to ground station to end user—could be typically smaller than approximately 10 s.

## Figures and Tables

**Table 1:** List of satellite (constellations) for an IoT/M2M service.

Mission	Institution/(Country)	Application/Service	Protocol	System Architecture	ISL	Data (IoT)	Latency	Frequency IoT	User terminal	Status	
ICARUS	Max Planck Society(DE)	Animal tracking	Proprietary	Sensor tag to ISS	no	UL 656 bps DL 520 bps	<day	402.25 MHz	5 g, 800 km TXPwr 6 mW	Operational 2019 Q3	
KEPLER	Kepler Comm. (CAN)	IoT Global data	Proprietary	140 × 3 U Cubesats	?	MB/month	<hour	?	?	Demonstrator: - 2 × 3U Cubesat: 2018 - 6U Cubesat: 2019 tbd	
FLEET	Fleet Space Tech. (AUS)	remote IoT	LoRaWAN	Sensor via gateway to LEO (Iridium), GEO (Inmarsat) Own fleet in dev	?	?	<min	to gateway: 902–928 MHz, 863–870 MHz, 433–434 MHz 802.11ac Wi-Fi	Range: 15 km #Ch.: 8 or 16 #nodes: < 1000 TXPwr 24 dBm	Demonstrator: - 3U Cubesat 2018.11	
HIBER	Hiber (NED)	IoT	?	48 sats 600 km SSO	?	1 msg (144 b)/day/node	<16 h	400 MHz	?	Demonstrator: - 2 Cubesats 2018 Q4	
KINEIS	Kineis (FRA)	IoT	?	20 nanosats by 2021	?	?	?	?	?	?	
Diamond	SAS Global (UK)	IoT Messaging	?	200 nanosats	yes	?	?	?	?	Demonstrator: - 3 × 3U 2017	

**Table 2:** Selected list of notable past and future small satellite ISL missions and parameters.

Mission	Org.(Nat)	S/C No.	Mass [kg]	ISL Payload	ISL Band	Data Rate [kbps]	Range [km]	Mission Purpose	Launch Status	Ref
SNAP-1	Surrey (UK)	2	6.5/49	-	UHF	9.6	2	tech demo	2000, success	
CanX-4&5	SSFTL (CAN)	2	5	-	S	10	5	PFF demo	2014, succ	[11]
EDSN	NASA (USA)	8	2	MHZ2420	UHF	9.6	20	multi-point measurement	2015, LV fail	[12]
Iridium Next	Lockheed (USA)	66	860	-	Ka	12,500	4500	mobile communication	2017, started	
Diamond	SAS (UK)	3	6	-	S	2000	-	ISL demo	2017, success	[13]
S-NET	TUB (GER)	4	9	SLINK	S	100	400	ISL demo	2018, success	[2]
CPOD	Tyvak (USA)	2	3	-	S	250	25	rendezvous, proximity	2020, expected	
PROBA-3	ESA (EU)	2	320/180	Gamalink	S	?	tbd	formation, autonomy	2019, expected	[14]

**Table 3:** Current terrestrial IoT protocols.

	NB-IoT	LTE-CATM1	EC-GSM-IoT	Sigfox	LoRa	RPMA	MIOTY
Modulation	OFDMA Half duplex	OFDMA Full duplex	TDMA, FDMA, GMSK, 8 PSK, Half Duplex	BPSK (EU) GFSK (USA) Pseudo-random freq. hopping	CSS modulation	DSSS, TDD frame structure	TS-UNB (Telegram-Splitting)
Frequency	typ. 800 MHz BW 180–200 kHz	typ. 1900 MHz BW 1.4 MHz	typ. 900 MHz	868 MHz (EU) 902 MHz (US)	868 MHz (EU) 915 MHz (US)	2.4 GHz ISM band	868 MHz (EU) 915 MHz (US) BW: 200–600 kHz
Data Rate	> 250 kbps typ. 100 kbps	> 1 Mbps typ. 384 kbps	70 kbps (GMSK) 240 kbps (8PSK)	100 bps	typ. 10 kbps	typ. 624 kbps	typ. 407 bps
Distance	LTE/GSM carrier range, 20 km	LTE/GSM carrier range, 25 km	GSM carrier range	<3–10 km city <30–50 km rural	<15 km city <50 km rural	25 km city 80 km rural	5 km city 15 km rural 30 km free space
Topology	star	star	star	star	star	star	star
#nodes	> 200 k	> 1 M	> 190 k	> 25 k	> 40 k	> 500 k	1.5 mio msgs/day or 500 k nodes (3 msgs/day)
TX power	20–23 dBm	20–23 dBm	23–33 dBm	14 dBm		21–30 dBm	14 dBm (EU), 16 dBm (US)
Link budget	164 dB	155.7 dB	160 dB	149 dB	157 dB	177 dB	154 dB (−140 dBm RX sensitivity)
Latency	1.5–10 s	10–15 ms	0.7–2 s	2 s	2 s	2.3 s	3.6–30 s (10–245 byte msgs)

**Table 4:** System design parameters for IoT/M2M applications and the affected OSI layers of an ISL link. A: Application, T: Transport, N: Network, D: Data Link, and P: Physical Layer.

System Parameter	Design	Affected OSI Layer					Simplex Narrow-Band IoT	Duplex Narrow-Band IoT	Duplex Gateway IoT	Space M2M	Real-Time IoT	
						Ground sensor→ satellite (ISL) → ground station	Ground sensor→ satellite (ISL) → ground station (+ backwards channel for sensor config/QoS)	Ground sensor→ gateway→ satellite (ISL) → ground station (+ backwards channel for sensor config/QoS)	Space sensor↔ satellite (ISL)→ ground station	Caller terminal↔ satellite (ISL)↔ responder terminal	**Link**
						·Freight tracking ·Smart metering ·Smart farming	·QoS applications ·Sensor config ·Infrastructure monitoring	·Smart metering ·Smart farming ·Infrastructure monitoring	·Distributed sensing with multiple satellites ·Planetary landers and probes ·Atmospheric science ·Gradiometry	·Voice communication ·Co-operative driving ·Near-real time messaging	**Application**
						·IoT (Position, sensor, status)	·IoT (Position, sensor, status) ·Config, QoS	·IoT (Position, sensor, status) ·Config, QoS	·Science ·Navigation ·Command ·Health and status	·Voice ·Message ·Config, QoS	**ISL Data**
Communication latency			N	D	P	min to hours	hours	hours	hours to days	near-real time	
Power ground terminal	A	T	N	D	P	ultra low (≪W)	ultra low (≪W)	moderate (∼W)	N/A	low (<W)	
Bandwidth Uplink				D	P	narrow (∼kbps)	narrow (∼kbps)	moderate (Mbps)	N/A	low (<Mbps)	
Onboard processing	A			D	P	high	high	high	high	very high	
Network topology	A	T	N	D	P	variable	variable	variable	variable	variable	
Connectivity	A	T	N	D	P	intermittent	intermittent	intermittent	intermittent	continuous	
ISL	A	T	N	D	P	maybe	maybe	maybe	yes	yes	
Reconfig./Scalability			N	D	P	high	high	high	high	high	

**Table 5:** Frequency bands for space-to-space communication.

Band	Frequency	Allocation Status
UHF	410–420 MHz	Space research (space-to-space)
	430–450 MHz	Amateur radio
L	1645.5–1656.5 MHz	Distress and safety communications
S	2025–2110 MHz	Space operations, Earth exploration, and space research services
	2200–2290 MHz	
Ku	13.4–13.65 GHz	Space research services
	14.5–15.35 GHz	
Ka	22.55–23.55 GHz	Intersatellite
	25.25–27.5 GHz	Space research services and Earth exploration

**Table 6:** Modulation types and data rates (in kbit/s) for TDD (from caller to responder).

Modulation	Distance		
	100 km	200 km	400 km
DBPSK, r=1/2	27.5	24.85	19.5
DBPSK, r=3/4	41.65	37.675	29.65
DQPSK, r=1/2	55.8	50.5	39.8
DQPSK, r=3/4	84.1	76.15	60.1
8-ADPSK, r=1/2	84.1	76.15	60.1
8-ADPSK, r=3/4	126.55	114.625	90.55
16-ADPSK, r=1/2	112.4	101.8	80.4
16-ADPSK, r=3/4	169.0	153.1	121.0

**Table 7:** Comparison of antenna concepts for ISL missions.

Feature	Single Antenna		Antenna Array
	Without Beam Forming	With Beam Forming	
Directivity	low	medium	high
Beam control	no	yes	yes
Attitude control	low	medium	high
Angular coverage	high	high	low
Occupied area	medium	medium	large
ISL range	low	medium	high
Complexity, cost	low	high	high
Medium access	TDMA, FDMA, CDMA	TDMA, FDMA, CDMA	TDMA, FDMA, CDMA, SDMA

**Table 8:** Overview of S-NET mission.

Parameter	Value
Number of satellites	4
Orbit height	580 km SSO
Launch date	2019/02/01
Design lifetime	1 year
Platform	TUBiX10
Mass	8.8 kg per S/C
Volume	25 × 25 × 25 cm^3^
TM/TC	UHF, TX power: 5 W
Power	Battery: Li-ion 5 Ah Solar cells: GaAs 30%
Attitude determination	<1 deg with MEMS array of sun sensor, gyro, magnetometer
Attitude control	<5 deg (3-axis) with 3× reaction wheels, 3× coils
Payload	SLINK: S band transceiver for ISL, UL, and DL Laser reflector for high precision position measurement
Ground station	UHF: Berlin, Backnang (Germany) and Svalbard (Norway) S band: Berlin

**Table 9:** Parameters of the SLINK radio.

Parameter	Value
Frequency ISL, DL	2210.2–2269.8 MHz
Frequency UL	2024.2–2109.8 MHz
Range ISL	100 km nominal, up to 800 km
Bit rate DL	0.674–3.394 Mbps
Bit rate UL	30.8–252 kbps
Bit rate ISL	8.8–126.55 kbps (DBPSK, 800 km; 8-ADPSK, 100 km)
Symbol rate ISL	80 kHz
RF bandwidth ISL	120 kHz
Multiplexing	TDD, P2P
Modulation	DBPSK, DQPSK, 8-ADPSK, and 16-ADPSK (optional)
Coding	Convolutional r=1/2,r=3/4
Decoding	Viterbi, soft decision
RF output	27 dBm (0.5 W)
Power	<12 W
Mass	<45 g with housing
Volume	140 × 80 × 65 ${\mathrm{mm}}^{3}$

**Table 10:** Link budget of S-NET ISL for various distances and pointing offsets.

	Scenario				Unit	Remark
ISL Distance	10 km	65 km	148 km	400 km		
Pointing Offset	45 °	45 °	30 °	0 °		
Frequency	2266	2266	2266	2266	MHz	
TX power output	27	27	27	27	dBm	
TX losses	1.5	1.5	1.5	1.5	dB	cable and connector
TX antenna gain	0	0	5	7.5	dBi	pointing offset
RX antenna gain	0	0	5	7.5	dBi	pointing offset
RX losses	1.5	1.5	1.5	1.5	dB	
RX sensitivity	−116.50	−116.50	−116.50	−116.50	dBm	
Roll-off factor	0.25	0.25	0.25	0.25	-	
Symbol rate	80	80	80	80	kbps	
RX noise factor	3.5	3.5	3.5	3.5	dB	
Link budget	140.50	140.50	150.50	155.50	dB	
Free space path loss	119.55	135.81	142.95	151.58	dB	
**Margin SNR**	**20.96**	**4.69**	**7.56**	**3.93**	dB	DBPSK+ CC r = 0.5
SNR required	4.00				dB	DBPSK+ CC r = 0.5
SNR required	6.00				dB	DBPSK+ CC r = 0.75
SNR required	9.00				dB	DQPSK+ CC r = 0.75
SNRrequired	13.00				dB	8ADPSK+ CC r = 0.75
SNR required	17.00				dB	16ADPSK+ CC r = 0.75

## Abbreviations

**ADPSK**	Adaptive Differential Phase Shift Keying
**ACK**	Acknowledge
**ARQ**	Automatic Repeat Request
**ADCS**	Attitude Determination and Control System
**AGC**	Automatic Gain Control
**ANS**	Antenna Search Algorithm
**AWGN**	Additive White Gaussian Noise
**BER**	Bit Error Rate
**BPSK**	Binary Phase Shift Keying
**BW**	Bandwidth
**CCSDS**	Consultative Cmte for Space Data Systems
**CC**	Convolutional Code
**CDMA**	Code Division Multiple Access
**COM**	Communication System
**CSS**	Chirp Spread Spectrum
**DL**	Downlink
**DBPSK**	Differential Binary Phase Shift Keying
**DQPSK**	Differential Quaternary Phase Shift Keying
**DSSS**	Direct Sequence Spread Spectrum
**EPS**	Electric Power System
**FDMA**	Frequency Division Multiple Access
**FEC**	Forward Error Correction
**GEO**	Geosynchronous Orbit
**GFSK**	Gaussian Frequency Shift Keying
**GMSK**	Gaussian Minimum Shift Keying
**GNSS**	Global Navigation Satellite System
**GSM**	Global System for Mobile Communications
**IoT**	Internet of Things
**ISL**	Inter Satellite Link
**ISM**	Industrial, Scientific and Medical Band
**ISS**	International Space Station
**LEO**	Low Earth Orbit
**LoRaWAN**	Low Range Wide Area Network
**LOS**	Line-of-sight
**LPWAN**	Low Power Wide Area Network
**LTE**	Long Term Evolution
**LV**	Launch Vehicle
**MAC**	Media Access Control
**MEMS**	Microelectromechanical Systems
**M2M**	Machine-to-Machine
**NAK**	Negative Acknowledge
**OSI**	Open Systems Interconnection Model
**OBC**	Onboard Computer
**OFDMA**	Orthogonal Frequency-Division Multiplexing
**PDH**	Payload Data Handling
**PSD**	Power Spectral Density
**PSK**	Phase-shift Keying
**P2P**	Point-to-point
**QoS**	Qualities of Service
**QPSK**	Quadrature Phase Shift Keying
**PFF**	Precise Formation Flying
**RF**	Radio Frequency
**RFD**	Responsive and Formal Design
**RPMA**	Random Phase Multiple Access
**RX**	Receive, Receiver
**S/C**	Spacecraft
**SDMA**	Space Division Multiple Access
**SDR**	Software Defined Radio
**SGP**	Simplified General Perturbations
**SNC**	Spacecraft Network Controller
**S-NET**	S-band network of distributed nanosatellites
**SNL**	Single Nanosatellite Launcher
**SNR**	Signal-to-Noise Ratio
**SSO**	Sun Synchronous Orbit
**TDD**	Time Division Duplex
**TDMA**	Time Division Multiple Access
**TM/TC**	Telemetry and Telecommand (Unit)
**TNC**	Terminal Node Controller
**TS-UNB**	Telegramm-Splitting – Ultra-Narrowband
**TUB**	Technische Universität Berlin
**TUBiX**	TU Berlin innovative neXt generation bus
**TX**	Transmit, Transmitter
**UHF**	Ultra High Frequency
**UL**	Uplink
**VHF**	Very High Frequency
**Pr**	Preambel (synchronisation field)
**Pkt**	Data packet (information field from network layer)
**Xmt**	Caller frame
**Rcv**	Responder frame
